# Application of whole-genome sequencing in a case study of renal tuberculosis in a child

**DOI:** 10.1186/s12879-020-4832-3

**Published:** 2020-02-05

**Authors:** Darja Aleinikova, Ilva Pole, Janis Kimsis, Anita Skangale, Olga Bobrikova, Regina Kazelnika, Inta Jansone, Inga Norvaisa, Iveta Ozere, Renate Ranka

**Affiliations:** 1 Latvian Biomedical Research and Study Centre, Ratsupites Str 1, k-1, Riga, LV-1067 Latvia; 2 Riga East University Hospital, Centre of Tuberculosis and Lung Diseases, Upeslejas, Stopiņi region, LV-2118 Latvia; 3 Madona Hospital, Rupniecibas Street 38, Madona, LV-4801 Latvia; 4 Riga Stradins University, 16 Dzirciema Street, Riga, LV-1007 Latvia

**Keywords:** Urogenital tuberculosis, Childhood tuberculosis, Genotyping, Whole-genome sequencing

## Abstract

**Background:**

Urogenital tuberculosis (TB) is rare in children and usually develops due to reactivation of the foci in the genitourinary tract after the latency period following initial infection. Urogenital TB in children has no pathognomonic clinical features that can result in overlooking or misdiagnosing this clinical entity. Here, we report important findings regarding the pathogenesis and transmission of TB by using genotyping and whole-genome sequencing (WGS) in a study of renal TB case in a child.

**Case presentation:**

A 13-year-old boy was admitted to the hospital because of high fever, severe dry cough, flank pain and painful urination. Abdominal ultrasonography and CT revealed an 8 mm calculus in the kidney, and clinical findings were initially interpreted as nephrolithiasis. Nevertheless, due to the atypical clinical presentation of kidney stone disease, additional investigations for possible TB were performed. The QuantiFERON®-TB Gold Plus test was positive, and the *Mantoux* test resulted in 15 mm of induration, confirming infection with *Mycobacterium tuberculosis* (Mtb). Chest X-ray was normal. Chest CT revealed calcified intrathoracic lymph nodes. The urine sample tested positive for acid-fast bacilli, and Mtb cultures were obtained from urine and bronchial aspirate samples, resulting in a final diagnosis of intrathoracic lymph node and renal TB. Contact investigation revealed that the child’s father was diagnosed with TB when the child was 1 year old. Genotyping and WGS analysis of Mtb isolates of the child and his father confirmed the epidemiological link and pointed to the latency of infection in the child.

**Conclusions:**

This case report confirmed the development of active TB from calcified lesions in adolescent after 12 years of exposure, demonstrated the absence of microevolutionary changes in the Mtb genome during the period of latency, and proved the importance of appropriate evaluation and management to prevent the progression of TB infection to active TB disease. The use of WGS provided the ultimate resolution for the detection of TB transmission and reactivation events.

## Background

Childhood tuberculosis (TB) occurs in the same world regions where adult TB is common. In 2017, the WHO estimated one million cases of childhood TB globally [1]; however, underdiagnosis, overdiagnosis and underreporting to national TB control programs interfere with the accurate evaluation of childhood TB epidemiology worldwide [2]. Children and adolescents represent clinically important populations with increased susceptibility to TB, and the risk of infection and disease after exposure to *Mycobacterium tuberculosis* (Mtb) is dependent upon host factors such as patient age and immune status [3]. Very young children have the highest risk of progressing to disease following infection, while children aged 5–10 years are somewhat protected until risk increases again in adolescence [4, 5].

Due to nonspecific and variable clinical symptoms, radiographic findings and paucibacillary nature of TB in children, physicians face serious challenges in confirming the diagnosis [6]. Some manifestations of extrapulmonary TB, such as TB meningitis and lymphadenitis, are observed more frequently in children and therefore are better described and recognized, while correct and rapid diagnosis of other forms could be more challenging [3].

Genotyping of Mtb is crucial for TB research and is widely used in studies of the Mtb strain population structure, exploration of pathogen evolution, its interaction with the human host and public health investigations, including confirmation of epidemiological links between patients (reviewed in [7]). Whole-genome sequencing (WGS) offers new opportunities both in research and public health applications by providing the ultimate resolution for strain classification to trace infectious sources and transmission networks and the prediction of the antimicrobial susceptibility profile of a given isolate (reviewed in [8]). The primary applications for WGS include diagnosis, treatment, surveillance and source investigation of Mtb infection, particularly species and subspecies identification, early determination of drug resistance patterns on the basis of the presence of specific SNPs and identification of transmission clusters and outbreaks [9].

Here, we report important findings regarding the pathogenesis and transmission of TB by using genotyping and WGS of Mtb in a study of intrathoracic lymph node and renal TB case in adolescent.

## Case presentation

A 13-year-old boy was admitted to the hospital because of complaints of high fever, severe dry cough, flank pain and painful urination. Fever and cough began 2 weeks prior to hospitalization, and flank pain and dysuria began 1 week prior hospitalization. Within 2 weeks of illness, the patient lost 8 kg of body weight; however, he still had a body mass index of 25 (height 151 cm, weight 58 kg). Prior to hospitalization, the patient had received amoxicillin and clarithromycin for 6 days; however, the symptoms did not diminish. In the hospital, treatment was continued with cefuroxime, and further investigations were conducted.

**Fig. 1:**
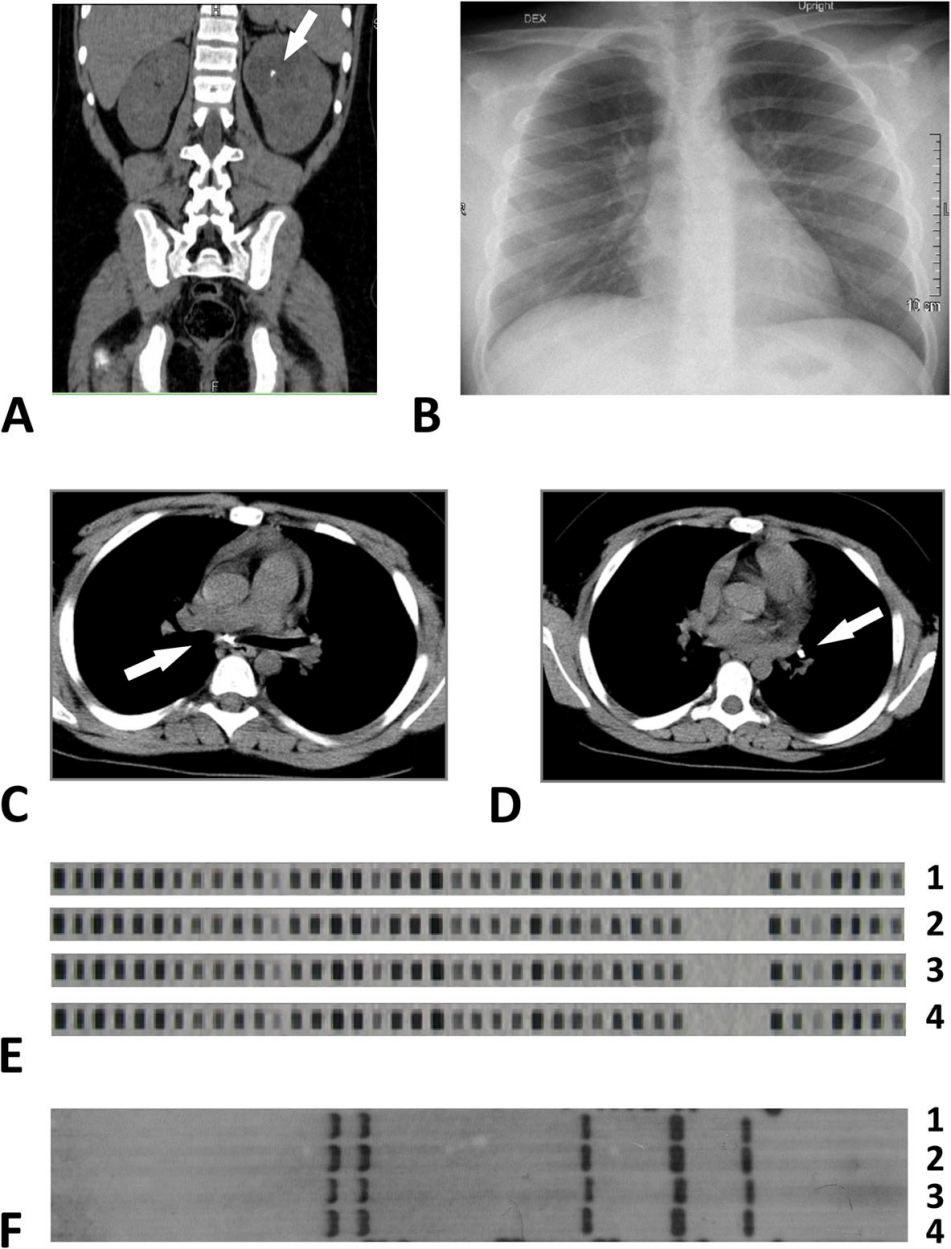
Pathological and genotyping findings. **a**: Abdominal computed tomography. A calculus in left kidney is shown (arrow). **b**: Chest X-ray. **c**, **d**: Chest computed tomography. Calcified intrathoracic lymph nodes in subcarinal and left bronchopulmonary lymph node groups (arrow). **e**: Spoligotyping analysis of Mtb isolates. Lane 1: urine sample, child (September 5, 2016); Lane 2: urine sample, child (September 6, 2016); Lane 3: bronchial aspirate sample, child (September 9, 2016); Lane 4: sputum sample, father (February 2, 2004). F: IS 6110 RFLP analysis of Mtb isolates. Lane 1: urine sample, child (September 5, 2016); Lane 2: urine sample, child (September 6, 2016); Lane 3: bronchial aspirate sample, child (September 9, 2016); Lane 4: sputum sample, father (February 2, 2004)

Blood tests showed elevated C-reactive protein levels (77.22 mg/L), elevated anti-streptolysin O antibody levels (1268.7 U/mL), and moderate hypochromic anemia. Urine analysis was normal prior to antibiotic treatment. Abdominal ultrasonography and computed tomography (CT) revealed a calculus 8 mm in size in the upper calyx of the left kidney and hydronephrotic transformation of the left kidney (Fig. 1 a). The patient’s blood parathormone level was low (< 0.32 pmol/L), but biochemistry analysis did not reveal any electrolyte abnormalities. Clinical findings were interpreted as nephrolithiasis, and lithotripsy was considered. Nevertheless, due to the atypical clinical presentation of kidney stone disease, additional investigations for possible TB were performed. BCG (Bacillus Calmette–Guérin) vaccination in the child was performed after birth with local scar formation of 5 mm in size. The QuantiFERON®-TB Gold Plus test was positive, and the *Mantoux* test resulted in 15 mm of induration, confirming infection with Mtb. Chest X-ray appeared normal (Fig. 1 b). Chest CT revealed calcified intrathoracic lymph nodes in subcarinal and left bronchopulmonary lymph node groups without lymph node enlargement in any group (Fig. 1 c, d). Nonsignificant interlobar pleural thickening in the right hemithorax and small calcified nodular shadow in the 8th segment of the left lung were additionally visualized on lung CT. In total, three urine samples were investigated for Mtb. Acid-fast bacilli (AFB) were positive on luminescence microscopy from the first urine sample, the Mtb DNA test by the GeneXpert® method was positive from the third consecutive urine sample, and cultures for Mtb were positive from the second and third urine sample. Bronchoscopy did not reveal any abnormalities in the tracheobronchial tree; however, Mtb was cultured on BACTEC medium from bronchial aspirate samples. In total, Mtb culture was positive from two urine and one bronchial aspirate samples. Mtb isolates were sensitive to isoniazid, rifampicin, ethambutol and levofloxacin. The final diagnosis was intrathoracic lymph node TB and renal TB.

At the time of TB diagnosis in the child, no infectious source case was identified. Family history revealed that in 2004, when the child was 1 year old (12 years ago), his father was diagnosed with pulmonary TB and had a positive sputum culture for Mtb*.* The child was examined and recognized as latently infected with Mtb; however, prophylactic treatment was not conducted. Father had received a full TB treatment course and was successfully cured. He passed away 2 years later of a different cause. Nevertheless, the father’s mycobacterial isolates were available from the mycobacteriology laboratory’s culture bank and regenerated, and genetic comparison of Mtb isolates of the child and his father was conducted.

Mycobacterial DNA was extracted from cultures grown on Löwenstein-Jensen media using the cetyltrimethyl ammonium bromide (CTAB) method [10]. All four isolates (one from father and three from the child) were included in the analysis. Spoligotyping was used to identify Mtb genotypes and was performed using commercially available kits (Isogen Life Science, Netherlands; later Ocimum Biosolutions, India) following a previously described standard protocol [11]. All isolates revealed the SIT53 spoligotype (Fig. 1 e). To prove the epidemiological link between the two disease cases, IS*6110* restriction fragment length polymorphism (IS*6110* RFLP) analysis was used. The IS*6110* RFLP pattern was identical for all four samples and represented with five bands (Fig. 1 f).

Furthermore, WGS analysis of Mtb DNA samples was performed. DNA was sheared physically using a Covaris S220 sonicator. Single-end fragment libraries were prepared using the IonPlus Fragment Library Kit (Thermo Fisher Scientific, US) according to the manufacturer’s protocol. Samples were sequenced on an Ion PGM™ system (Thermo Fisher Scientific, US), and reads of a maximum length of 400 base pairs were produced. Bioinformatic analysis of sequencing data was performed on the Galaxy web platform using the public server at https://usegalaxy.org [12]. Briefly, filter by quality (v 1.0.2) was used to keep only those sequences whose Phred quality score was at least 10 for 95% of nucleotides and at least 20 for 80% of nucleotides. Reads were trimmed for adapter content and low-quality ends (Phred quality score < 20) using Trim Galore! (v0.4.3.1), keeping sequences longer than 30 base pairs. Low-quality reads and reads of inappropriate length (longer than 400 bp) were then discarded by Filter FASTQ (v1.1.1), and the final output was mapped to the genome sequence of Mtb strain H37Rv (GenBank NC000962.3) using Bowtie2 (v2.3.4.2). Reads laying within highly variable repetitive regions (PE/PPE genes) were excluded by BAM filter (v 0.5.9), and duplicate reads were removed using Picard tools (MarkDuplicates, v2.18.2.1). All genome sites were called using SAMtools mpileup (v2.1.4), and single nucleotide polymorphisms (SNPs) were detected with VarScan (v2.4.2). Mutations within PE/PPE genes were removed using Slice VCF (v 0.1), and SNPs at the sites of unusual depth were ignored. Each identified SNP had to be supported by at least 4 reads representing both forward and reverse strands with base quality scores greater than 20. Position was considered homozygotic if more than 75% of reads supported the variant. All SNP differences between analyzed samples were checked manually using Integrative Genomics Viewer (IGV, v2.5.3). For all four sequenced isolates, more than 98% of the Mtb H37RV genome was covered with a mean depth of more than 40 reads per base (Table 1).

**Table 1:** Analysis of *Mycobacterium tuberculosis* isolates by whole genome sequencing

	*Mycobacterium tuberculosis* isolates			
	Father	Child		
Isolation source	Sputum	Urine	Urine	Bronchial aspirate
Isolation date	February 4, 2004	September 5, 2016	September 6, 2016	September 9, 2016
Genome coverage (%)^a^	98.28	99.13	98.22	98.38
Average read length (bp)	213	173	205	188
Mean base quality	31.1	30.7	30.6	30.7
Mean depth	40.4	61.5	40.3	62.8

^a^ *Mycobacterium tuberculosis* H37RV genome

The results showed that the genetic distance between the father’s sputum Mtb isolate and the child’s bronchial aspirate sample was only 1 SNP. This SNP (G/A, position 2,838,120, coverage depth: 75 reads, mapping quality: 42, mean base quality: 30) was homozygotic and supported by 100% of covering reads. No differences between father’s sputum Mtb isolate and child’s isolates obtained from either urine portion were observed. These findings confirmed the epidemiological link between the two TB cases and pointed to the latency of infection in the child.

## Discussion and conclusions

The use of molecular epidemiology in our case study revealed several important aspects of childhood renal TB pathogenesis, prevention, clinical manifestation, and transmission. During initial pulmonary infection, Mtb spreads via the lymphohematogenous route and can reach any organ and system, including the kidneys. Renal TB usually develops due to reactivation of the TB foci in the genitourinary tract after the latency period ranging from 5 to 10 and even 40 years after initial infection [13]. However, renal TB can also be a part of congenital TB in newborns and infants or miliary TB without age limitation [14]. In our case study, intrathoracic lymph node TB and renal TB were diagnosed in a 13-year-old boy. Infectious source case tracing and results of two genotyping methods, i.e., spoligotyping and IS*6110* RFLP, pointed to the father of the child as a possible source of infection. Further application of Mtb WGS confirmed that in our patient, TB infection progressed to urogenital and respiratory TB 12 years after exposure. These findings also highlighted the importance of the maintenance of the culture bank and access to Mtb TB samples and clinical information from previous TB cases. This appeared to be especially useful in our case study, allowing confirmation of exposure to TB sources, which occurred more than a decade before TB disease developed in our patient.

WGS is commonly used worldwide for Mtb outbreak investigation and for determination of possible transmission roots between patients. This approach is based on the detection of strain microevolution during outbreaks and the assessment of accumulated SNPs in clinical isolates. The mutation rate of Mtb of approximately 0.3–0.5 SNPs per year have been calculated in several outbreak studies [15, 16]. However, the mutation rate during the latent state of infection remains unclear, as data from studies differ: the results show that during latency, Mtb mutates either at a similar rate or approximately ten times slower than in the active state [17–20]. In our study, WGS analysis did not reveal any differences between father’s sputum Mtb isolate and child’s urine isolates, while the genetic distance between father’s sputum Mtb isolate and child’s bronchial aspirate sample was only one SNP. These findings provided additional confirmation of the epidemiological link between those two TB cases. In addition, the absence of microevolutionary changes in the Mtb genome during the 12-year period indirectly supports the evidence of latency of TB infection in the child.

The prechemotherapy literature suggests that without prophylactic treatment, primary infection in children younger than 2 years of age frequently (even up to 50% of cases) progresses to TB disease, including severe forms such as meningitis and miliary TB [5]. Adolescence represents the second high-age-risk period during which primary infection progresses to TB disease with a frequency of 10–20% and is associated with the development of adult-type disease [3].

In our patient, the primary TB infection at the time of exposure at 1 year of age did not progress to clinically severe disease, probably due to the protective effect provided by BCG vaccination at birth. Primary infection apparently resulted in calcified lesions within intrathoracic lymph nodes and left kidney, and TB reactivation occurred during adolescence. Calcified lesions are commonly considered healed TB lesions with minimal risk of disease progression. On the other hand, bacilli can persist in a latent stage in a small percentage (approximately 1%) of these evolved lesions and can resuscitate after some type of local immunosuppression and produce active TB [21]. Our findings demonstrated the risk of reactivation of untreated latent TB infection from calcified lesions. Interestingly, despite calcified (but not enlarged) lymph nodes in the mediastinum and small calcified nodulus in the lung parenchyma, the child had a positive culture for Mtb from a bronchial aspirate sample, indicating the presence of live Mtb in his respiratory tract.

The importance and necessity of prophylactic treatment and the availability of effective monitoring tools, including biomarkers of TB progression, should also be considered. The benefits of prophylactic treatment of TB infection have been known for more than 60 years, and the risk reduction for developing active TB was shown in children aged 15 years and younger [22]. Since 2012, WHO guidance recommends the use of prophylactic treatment for children younger than 5 years who are household contacts of infectious pulmonary TB patients [23]. In 2018, this guidance was updated to include the option for preventive therapy for older children with a positive tuberculin skin test [24]. Additionally, the END TB strategy calls for appropriate household contact management and prophylactic treatment of persons at high risk for TB disease [25]. Unfortunately, our patient did not receive prophylactic treatment of latent TB infection after exposure, which could have averted the development of TB during adolescence. This case report highlights the importance of appropriate TB contact management and strongly supports the guidelines to improve the prophylactic TB treatment rates among the childhood population.

In conclusion, this unique case report confirmed the development of active TB from calcified lesions in adolescent after 12 years of exposure, demonstrated the absence of microevolutionary changes in the Mtb genome during the period of latency, and proved the importance of appropriate evaluation and management after TB exposure to prevent progression to active TB disease. The use of WGS analysis provided the ultimate resolution for the detection of TB transmission and reactivation events.

## Data Availability

The datasets generated and analysed during the current study are available from the corresponding author on request.

## Abbreviations

BCG: Bacillus Calmette–Guérin
CT: Computed tomography
Mtb: *Mycobacterium tuberculosis*
RFLP: Restriction fragment length polymorphism
SNP: Single nucleotide polymorphism
TB: Tuberculosis
WGS: Whole genome sequencing
WHO: World Health Organization
